# Dichotomous effects of Galectin-9 in disease modulation in murine models of inflammatory bowel disease^☆^

**DOI:** 10.1016/j.biopha.2025.117902

**Published:** 2025-03

**Authors:** Samantha Tull, Anella Saviano, Areeba Fatima, Jenefa Begum, Adel Abo Mansour, Noemi Marigliano, Anna Schettino, Julie Blaising, Patrick Trenkle, Virginie Sandrin, Francesco Maione, Daniel Regan-Komito, Asif J. Iqbal

**Affiliations:** a Department of Cardiovascular Sciences (CVS), College of Medicine and Health, University of Birmingham, Birmingham B15 2TT, UK; b ImmunoPharmaLab, Department of Pharmacy, School of Medicine and Surgery, University of Naples Federico II, Via Domenico Montesano 49, Naples 80131, Italy; c Department of Clinical Laboratory Sciences, College of Applied Medical Sciences, King Khalid University, Abha, Saudi Arabia; d Roche Pharma Research & Early Development, CMV, Immunology, Infectious Diseases and Ophthalmology (CMI2O), Roche Innovation Center Basel, F. Hoffmann-La Roche Ltd, Grenzacherstrasse 124, Basel 4070, Switzerland

**Keywords:** Galectin, IBD, Monocyte, Macrophage, T cell

## Abstract

Inflammatory bowel disease (IBD) is a multifaceted disease characterised by compromised integrity of the epithelial barrier, the gut microbiome, and mucosal inflammation. While leukocyte recruitment and infiltration into intestinal tissue are well-studied and targeted in clinical practice, the role of galectins in modulating mucosal immunity remains underexplored. Galectins, a family of lectin-binding proteins, mediate critical interactions between immune cells and the intestinal epithelium. This study investigated the effect of endogenous Galectin-9 (Gal-9), as well as the combined effects with Galectin-3 (Gal-3), in modulating disease progression in murine models of colitis, using global knockout (KO) models for Gal-3, Gal-9, and Gal-3/Gal-9. Global deficiency in both galectins demonstrated improved disease parameters in Dextran sodium sulfate (DSS)-driven colitis. In contrast, in a model of adoptive T cell driven colitis, the addition of recombinant Gal-9 (rGal-9) was associated with reduced intestinal inflammation and an improvement in disease parameters. Further *in vitro* studies revealed no change in bone marrow-derived macrophage cytokine production in the absence of endogenous Gal-9, whereas the addition of rGal-9 to human macrophages stimulated pro-inflammatory cytokine production. Collectively, these findings demonstrate that Gal-9 plays distinct, context-dependent effects in intestinal inflammation, with both pro-inflammatory and anti-inflammatory effects. The contrasting functions of endogenous and exogenous Gal-9 underscore its complex involvement in IBD pathogenesis and highlight the need to differentiate its physiological function from therapeutic applications.

## Highlights

• Gal-9 deficiency reduces inflammation in DSS-induced colitis in mice.
• Recombinant Gal-9 treatment in T cell adoptive transfer model reduces colitis.
• Gal-9's impact on inflammation is context-dependent and cell-specific.
• Targeting Gal-9 offers potential therapeutic strategies for IBD.

## Graphical Abstract

**Figure:**
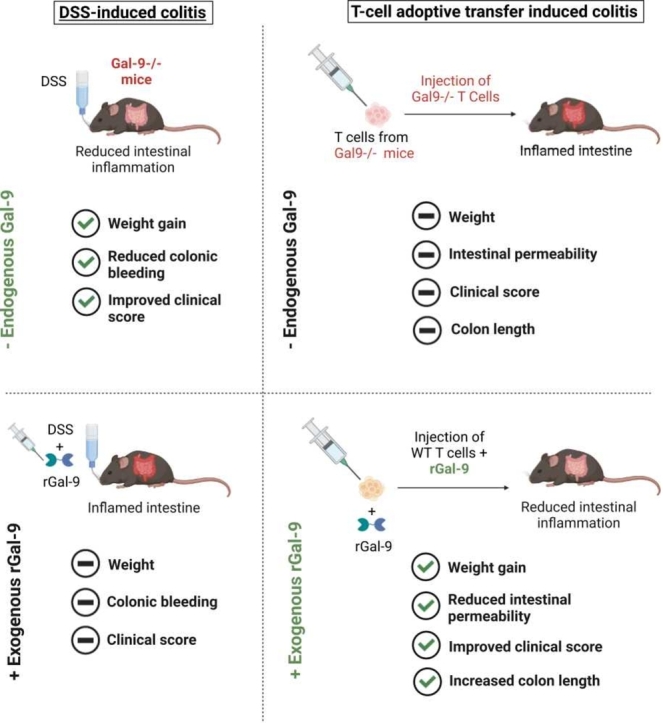

## 1 Introduction

The holistic health of the gut is characterised by the integrity of the epithelial barrier, the microbiome, and its mucosal immune cells, which are maintained by an intricate network of immune signalling events involved in removing harmful pathogens without triggering host cell damage or persistent inflammation [1], [2]. With the majority of human immune cells located within the gut, recruitment of circulating leukocytes, and subsequent infiltration, is a critical component in regulating intestinal homeostasis. Since this response is self-limiting during episodes of acute inflammation, unresolved and dysregulated leukocyte infiltration remains central to the pathogenesis of chronic inflammatory conditions, such as IBD [1], [3], [4]. Hence, factors which modulate leukocyte function and recruitment are desirable therapeutic targets for patients with IBD, including ulcerative colitis (UC) and Crohn’s Disease (CD).

Lectins are amongst the key endogenous mediators involved in leukocyte trafficking to the intestinal mucosa and are regulated by host environment, inflammation and metabolism [5], [6]. Aberrations to glycan interactions, can lead to altered mucosal immunity which is implicated in the pathogenesis of IBD [7], [8], [9]. Galectins, a family of β-galactoside-binding proteins, have been identified as key modulators of immune cell function both during homeostatic and inflammatory states, where they influence chemokine production, apoptosis, cell adhesion, and chemotaxis [1], [10]. Their ability to promote cross-linking, reorganisation, and clustering of the glycosylated receptors resulting in increased control over immune cell activation and signalling. This is second to their extensive immunomodulatory capabilities via their control over innate and adaptive immune cell activation and maturation, as well as cytokine receptor expression [1], [11], [12].

Specifically, in IBD patients, altered expression of galectins within the gastrointestinal tract and in serum has been reported. Gal-1 and Gal-3 serum levels have been shown to be elevated in IBD patients compared to healthy controls [13]. In contrast, other members of the galectin family, specifically Gal-9 have emerged as a significant player in immune regulation and suppression. As a member of the tandem repeats galectin family, Gal-9 consists of a single polypeptide chain with two unique carbohydrate recognition domains CRD linked by a small peptide [5], [10]. Widely expressed across multiple tissue types and species, the expression of Gal-9, specifically in the mucosa, is observed on the epithelial cells of the small and large intestine [14]. Gal-9 has been shown to exhibit both pro-inflammatory and anti-inflammatory effects in varying contexts of disease [10], [15]. Studies looking at human sera, and supernatants from epithelial cells display increased levels of Gal-9 during infection and inflammation [16], [17]. A protective effect for Gal-9 has also been implicated whereby global deletion of endogenous Gal-9 showed increased sensitivity to chemically induced colitis and multiple models of epithelial injury. Similarly, in humans, increased levels of Gal-9 were associated with enhanced intestinal crypt regeneration in patient biopsies, suggesting a conserved mechanism [18]. Additionally, exogenous Gal-9 can induce apoptosis in Th1 and Th17 cells, both of which are subsets known to contribute to the inflammatory milieu and pathogenesis in inflammatory diseases such as IBD and rheumatoid arthritis (RA) [1], [19], [20], [21]. Further studies have demonstrated the ability of exogenous Gal-9 to promote inflammatory resolution through the expansion of regulatory T cell (Treg) populations, promoting immune tolerance and reduced inflammation [10], [19], [22]. Gal-3, on the other hand, is the only member of the chimera-type classification, which consists of a C-terminal CRD and a large non-lectin N-terminal domain, and can engage with enteric bacteria and impact their colonisation [5]. Studies have indicated that Gal-3 exhibits a predominantly pro-inflammatory effect on intestinal inflammation driven by activation of the NLRP3 inflammasome and IL-1β production [23].

Dysregulation in T-cell responses is widely recognised in IBD patients with distinct patterns of immune cell recruitment reported in both UC and CD [24]. Historically, it was presumed that both could be clearly defined by the specific recruitment of T helper cells (Th), where increased activation of mucosal Th1 and/or Th17 cells were noted in patients with CD, whereas the gut of UC patients remained partial to Th2 recruitment [25], [26]. However, emerging evidence indicates that this distinction is more complex than previously understood [27]. Since Th cells are responsible for the downstream recruitment of B-cells, T-cells, and macrophages, their potential influence via galectin activity remains an active area of interest [25]. With their effects not restricted only to T cells, galectin activity and its behavioural changes to macrophages have also been explored. Excessive infiltration of macrophages into inflamed mucosa marks a characteristic feature of IBD [28], [29], [30].Studies with peritoneal macrophages indicate that macrophages which lack Gal-3 display reduced phagocytic capacity of apoptotic cells, compared to wildtype (WT) macrophages, resulting in delayed inflammatory resolution [10]. Whereas Gal-9 has been shown to promote an M2 anti-inflammatory macrophage phenotype in human macrophages [31].

Building on the critical functions of galectins in regulating immune responses and maintaining intestinal homeostasis, this study investigates the effects of galectins in regulating immune responses and maintaining intestinal homeostasis, with a focus on the specific functions of Gal-3 and Gal-9 in IBD. Using murine models of colitis and *in vitro* macrophage activation assays, we aim to uncover the effects of these galectins in both regulating and driving inflammation. By exploring their influence on T-cell responses, macrophage activity, and epithelial repair in IBD, this study seeks to identify novel therapeutic approaches to tackle the critical challenges posed by UC and CD.

## 2 Methods

### 2.1 Animals

All animal care and experimental procedures complied with reporting of *in vivo* experiments (ARRIVE) guidelines, international and national law and policies and were approved (Authorisation number: 533/2021-PR) by the Italian Ministry of Health (EU Directive 2010/63/EU for animal experiments, and the Basel declaration including the 3Rs concept). Male BALB/c and immunodeficient RAG1 KO were purchased respectively from Charles River (Milan, Italy) and Jackson Laboratories (Bar Harbon, USA). Galectin-9 knockout mice (Gal-9 KO) B6(FVB)-*Lgals9*^*tm1.1Cfg*^/Mmucd, RRID:MMRRC_031,952-UCD, were obtained from the Mutant Mouse Resource and Research Center (MMRRC) at University of California at Davis, an NIH-funded strain repository, and was donated to the MMRRC by Jim Paulson, Ph.D., The Scripps Research Institute. Gal-3 KO mice were purchased from Charles River, UK. Animals were housed under specific pathogen-free conditions in ventilated cages under controlled temperature and humidity, on a 12 h light/dark cycle and allowed *ad libitum* access to standard laboratory chow diet and sterile water. Experimental study groups were randomized, and their assessments were carried out by researchers blinded to the treatment groups.

### 2.2 DSS-induced colitis model

DSS colitis experiments were performed at Fidelta Ltd., Croatia. Animals were transferred to Fidelta Ltd and were allowed 2 weeks to acclimatise before the experiment began. Murine DSS-induced colitis was induced by administration of 3 % DSS in drinking water daily for 7 days in WT, Gal-9 KO, Gal-3KO, and Gal-9/Gal-3 double KO mice, whilst the control group received water only. In some experiments Gal-3 (50 µg) or −9 (50 µg) were administered daily (total of 7 days) intraperitoneal to WT mice. In-life surrogate markers of intestinal inflammation were recorded for each mice group immediately prior to DSS treatment and daily for 7 days post DSS administration. Daily body weight was recorded and used to calculate percentage change in body weight over time. Stool consistency and colorectal blood were also recorded. Disease activity index (DAI) scores were calculated using body weight change score, stool consistency score, and colorectal bleeding score. These parameters were given a severity score, ranging from 0 to 4. A sum of these three scores produced a disease activity (DA) index score, ranging from 0 to 12.

### 2.3 T-cell transfer model of colitis

T cell transfer colitis experiments were performed at Epistem Ltd, U.K or University of Naples Federico II, Italy . Adoptive transfer of CD4^+^CD45RB^high^ T cells (naïve T cells) from healthy donor mice (BALB/c) into RAG1 KO recipient mice induced colitis approximately 4–7 weeks following the T cell transfer. Histopathological inspection of the colon from mice with active disease reveals similar pathology findings in IBD patients [32], [33]. CD4^+^ T cell subsets were isolated from the spleen of BALB/c mice as described previously [34]. Briefly, a single-cell suspension was prepared, smashing spleens through a 40 μm cell strainer and lysing red blood cells with ACK buffer [35]. CD4^+^ T cells were first isolated using the anti-CD4 (L3T4)-MACS system (Miltenyi Biotec, Germany) according to the manufacturer's instructions. Enriched CD4^+^ T cells (96–97 % pure) were then labelled with CD4 (1 μg ml^−1^; clone RM4–5, c.n.: 17–0042–82), CD25 (1 μg ml^−1^; clone 7D4, c.n.: 558642) and CD45RB (2.5 μg ml^−1^; clone 16 A, c.n.: 553100) antibodies (all from BD Biosciences) for 20 min at 4 °C. CD4^+^CD25^−^CD45RB^high^ cells fraction was purified, under sterile conditions, using a FACS Aria Fusion cell sorter (BD Biosciences, California, USA) [36], [37]. The purity of cell isolates was confirmed by flow cytometry analysis. The CD45RB^high^ and CD45RB^low^ populations were defined as the brightest staining 40–60 %, and the dullest staining 15–20 % CD4^+^ T cells, respectively [34], [38], [39]. Thereafter, each recipient mouse was injected intraperitoneally (i.p.) with 0.5 × 10^6^ naïve colitogenic T cells in 500 μl of sterile PBS [40], [41] and the body weight was monitored three times weekly for 7 weeks, according to the procedures previously described [38], [39], [42]. Purified cells were injected into WT or Gal-9 KO Rag^(-/-)^ recipient mice with or without Gal-9 supplementation (3 µg/ml day 1, 3 & 5, intraperitoneal). In-life markers of intestinal inflammation were assessed in Rag^(-/-)^ mice from each group on days 0–7 following adoptive transfer of donor CD4^+^ T cells. In-life markers included body weight and % faecal water content. *Ex-vivo* tissue markers of inflammation measured at the end of the study, including evaluated colonic length, and overall clinical scores. All qualitative assessments of inflammation were performed blind.

### 2.4 Clinical observation and intestinal permeability assay

All mice were observed for clinical signs and, at autopsy, the clinical score was assessed by three investigators who were blinded to the experimental conditions. Clinical score was assessed by the sum of three parameters as follows: hunching and wasting, 0 or 1; colon thickening and/or inflammation score (0, no colon thickening and/or no inflammation; 1, mild thickening and/or local hyperaemia without ulceration; 2, moderate thickening and/or ulceration without hyperaemia; 3, extensive thickening and/or one or more sites of ulceration and inflammation); and stool consistency score (0, normal beaded stool; 1, soft stool; 2, diarrhoea; 3, bloody stool) accordingly to previous studies [37], [43]. For intestinal permeability assay, mice were denied access to food but allowed water for 3 hrs before gavage with 0.2 ml of PBS (pH 7.49) containing 22 mg/ml permeability tracer dextran (c.n.: 4013, Chondrex, Inc., Woodinville, USA). Blood samples were obtained by an intracardiac puncture after 3 hrs and centrifugated (1000 g, at 4 °C) for 20 mins. For use as measurement, harvested sera (light protected), mixed with an equal volume of PBS, freshly prepared standards as well as blanks (PBS and diluted sera from untreated animals) were transferred to a black 96-well microplate. Analysis of dextran was carried out with a fluorescence spectrophotometer (GloMax® Explorer microplate reader, Promega) using excitation wavelengths of 490 nm and emission wavelengths of 520 nm [39].

### 2.5 Murine bone marrow derived macrophage (BMDM) isolation and stimulation

Cells from murine bone marrow were extracted from tibiae and femurs of WT and Gal-9 KO mice (C57BL/6) (5 mice per group). Isolated cells were cultured in RPMI 1640 (Gibco) supplemented 2 mM L-glutamine, 10 % heat-inactivated fetal bovine serum (FBS), 1 % penicillin-streptomycin (100 U/ml penicillin, 100 μg/ml streptomycin) and 50 ng/ml macrophage-colony stimulating factor (MCSF) for 5 days in 100 mm x 20 mm cell culture-treated petri dishes (Corning) at 37 °C in 5 % CO₂. On day 6, BMDMs were gently lifted from petri dishes using cell dissociation buffer (Gibco) at 37 °C for 20 mins. Harvested cells were re-seeded into 12 well tissue-culture treated plates (Corning) in media containing RPMI 1640 and 1 % penicillin-streptomycin at a density of 1 × 10^6^ cells per well [44]. BMDMs were stimulated on day 7 with either LPS (100 ng/ml)/IFN (20 ng/ml) PolyI:C (25 μg/ml) and zymosan 10 μg/ml for 16 hrs and the resulting supernatants were collected and measured for cytokine release via ELISA or proteome profile arrays.

### 2.6 ELISA

Cytokine release from BMDM cell culture supernatants were measured for IL-6, TNFα, and IL-10 using Quantikine ELISA assays (R&D systems) according to manufacturer’s instructions.

### 2.7 Proteome microarray

Cell culture supernatants from BMDMs were pooled together for multiplex analysis of a range of 36 cytokines using a pre-set proteomic profiler human microarray according to manufacturer’s instructions (R&D systems).

### 2.8 Luminex

Cytokine levels in cell culture supernatants from human monocyte-derived macrophages were measured via Luminex.

### 2.9 Human monocyte-derived macrophage culture and stimulation

Frozen CD14^+^ monocytes were brought up into culture (RPMI 1640, 10 % FBS, 50 ng/ml MCSF) for 5 days and differentiated in 100 ng/ml MCSF (Peprotech). At day 5 differentiated macrophages were harvested and seeded into 96 well tissue-culture treated plates at a seeding density of 5 × 10^4^ cells per well. The following day macrophages were pre-treated for 20 mins with varying concentrations of Gal-3 and Gal-9 (0, 10, 30, 100, 300 nM) in the presence or absence of LPS (100 ng/ml). Culture supernatants were harvested after 16 hrs and analysed for cytokine levels via Luminex.

### 2.10 Statistical analysis

Statistical analysis complies with the international recommendations on experimental design and analysis in pharmacology and data sharing and presentation in preclinical pharmacology. Data are presented as mean ± S.D. Normality was tested prior to analysis with one or two-way ANOVA followed by Bonferroni's or Dunnett's for multiple comparisons, where P ≤ 0.05 was deemed significant. GraphPad Prism 9.0 software (San Diego, CA, USA) was used for analysis. For *in vivo* studies, animal weight was used for randomization and group allocation to reduce unwanted sources of variations by data normalization. No animals and related *ex vivo* samples were excluded from the analysis. The study was carried out to generate groups of equal size (n = 6 of independent values) using randomization and blinded analysis.

## 3 Results

### 3.1 Systemic Gal-9 and Gal-3 deficiency were associated with a reduction in in-life surrogate markers of intestinal inflammation

**Fig. 1:**
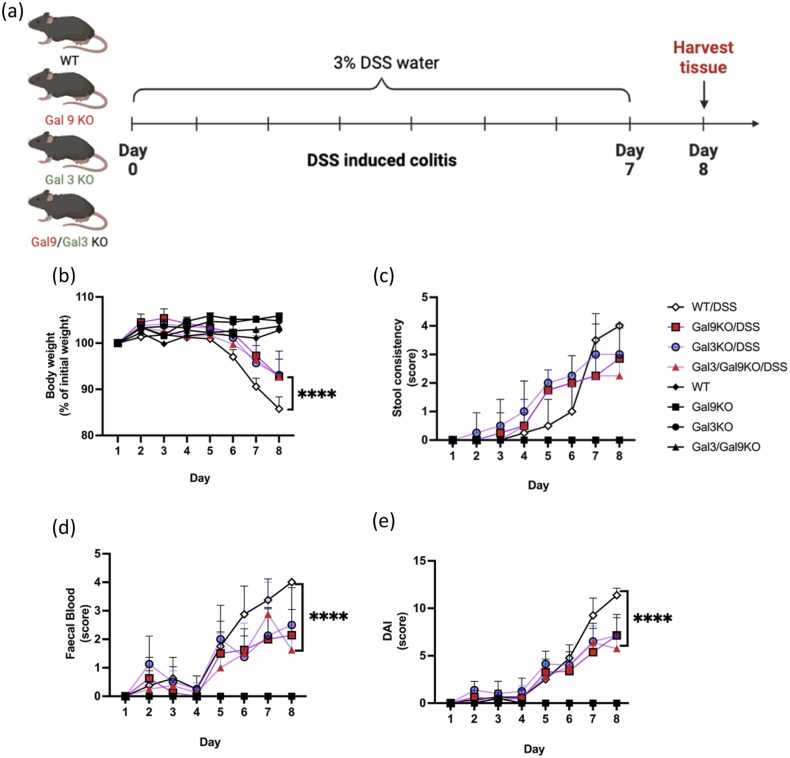
**Systemic Gal9 and Gal3 deficiency reduced intestinal inflammatory parameters in DSS-induced model of colitis.** (A) Schematic representation of experimental DSS-induced colitis model was induced by daily administration of 3 % DSS in WT, Gal-9 KO, Gal-3 KO, Gal-9/Gal-3 double KO mice for 7 days followed by harvesting of tissue on day 8. (B) Changes in body weight (expressed as percentage of initial weight) (C) stool consistency, (D) faecal blood score and (E) disease activity index (DAI) was recorded over the 7 days of the experimental model. Data are represented as means ± S.D (n = 2–8). Statistical analysis was performed using two-way ANOVA followed by Dunnett’s for multiple comparisons post-hoc test; *P ≤ 0.05, ^**^P ≤ 0.01, ^***^P ≤ 0.001, ^****^P ≤ 0.0001.

Initially, we sought to investigate the effect of endogenous Gal-9 and Gal-3 deficiency in a model of acute intestinal inflammation. For this C57BL/6 WT, Gal-9 KO, Gal-3 KO and combined Gal-9 and Gal-3 KO mice were subjected to Dextran Sodium Sulfate (DSS) induced colitis causing severe intestinal inflammation driven by epithelial cell damage (Fig. 1A). Effects of Gal-9 or Gal-3 deficiency were assessed for the following disease-associated parameters, including body weight, stool consistency, faecal blood scores (indicative of colonic bleeding) and DAI scores. Body weight decreased in all DSS-treated groups whilst remaining stable in untreated WT and KO mice over the study period, with no significant changes between the groups. Interestingly, the deficiency of Gal-9 and Gal-3 alone appeared to reduce DSS-associated weight loss (% body weight), colonic bleeding (faecal blood scores) and improve overall DAI scores, all to a similar extent by day 8 compared to WT DSS-induced control mice. Similarly, these findings were replicated with both Gal-9 and Gal-3 double KO, suggesting a potential pro-inflammatory effect of both galectins during intestinal inflammation (Fig. 1 B-E).

**Fig. 2:**
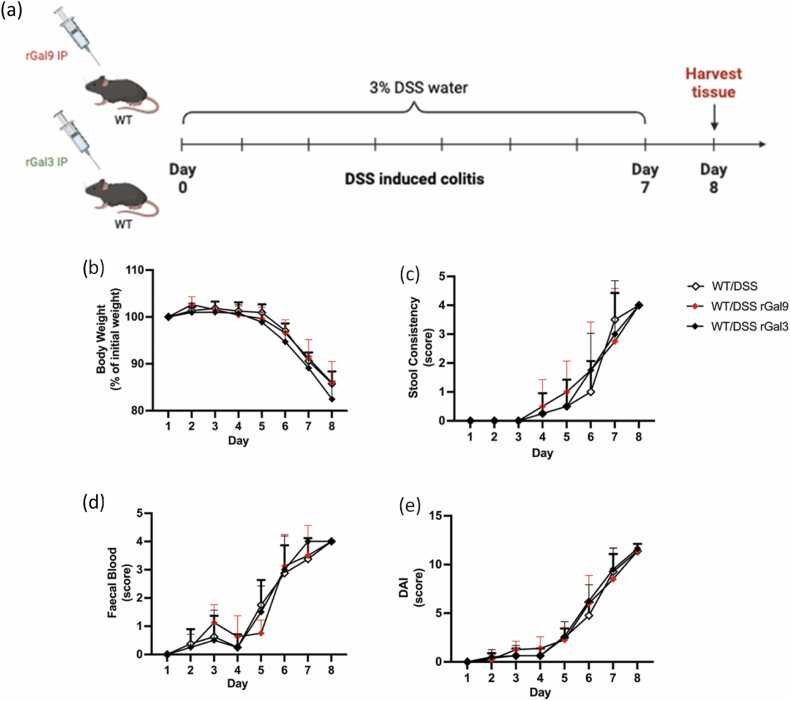
**Exogenous Gal9 had no effect on intestinal inflammatory parameters in DSS-induced model of colitis.** (A) Schematic representation of WT mice supplemented daily with recombinant intraperitoneal Gal-9 (50 µg) and Gal-3 (50 µg) which were subject to DSS-induced colitis by the administration of 3 % DSS for 7 days, followed by tissue harvest on day 8. (B) Changes in body weight (expressed as percentage of initial weight) (C) stool consistency, (D) faecal blood score and (E) disease activity index (DAI) was recorded over the 7 days of the experimental model. Data are represented as means ± S.D (n = 2–8). Statistical analysis was performed using two-way ANOVA followed by Dunnett’s for multiple comparisons post-hoc test; *P ≤ 0.05, ^**^P ≤ 0.01, ^***^P ≤ 0.001, ^****^P ≤ 0.0001.

In our initial pilot experiments, we tested recombinant Gal-9 (rGal-9) and recombinant Gal-3 (rGal-3) at 3 µg and 20 µg in the DSS model but observed no differences. Therefore, we decided to conduct a more powered study using a higher dose of 50 µg. Despite this, the addition of rGal-9 (50 µg) or rGal-3 (50 µg) did not result in any additional changes in DSS-induced disease severity. We did not observe any significant differences across the disease-associated parameters (Fig. 2 A-E), indicating that endogenous Gal-9 and Gal-3 contribute to driving disease-related inflammatory responses in this colitis model. Thus, both galectins may represent potential therapeutic targets in colitis-associated inflammation.

### 3.2 Exogenous Gal-9 treatment reduced T-cell driven intestinal inflammation

**Fig. 3:**
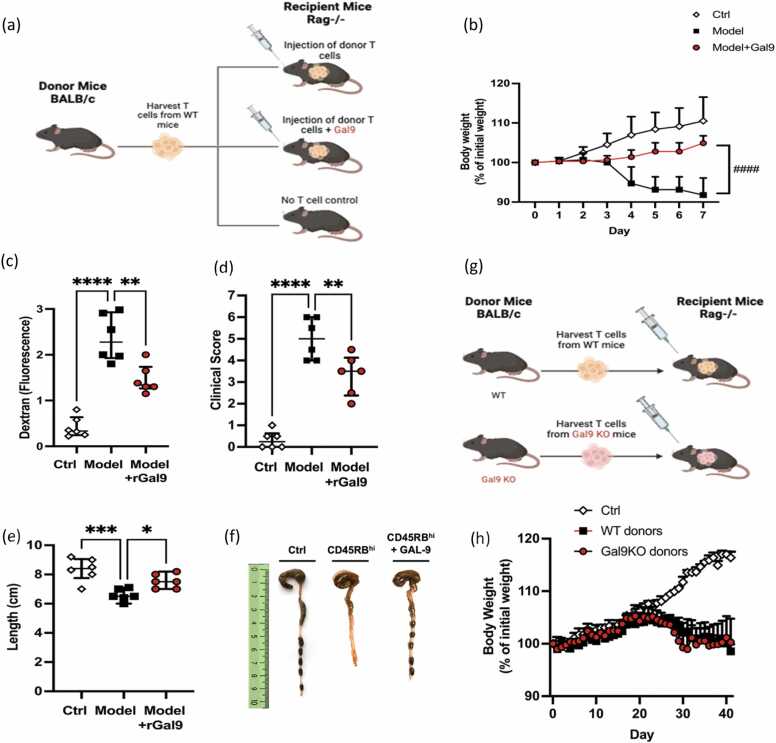
**Effects of Gal-9 on T-cell driven intestinal inflammation.** (A) Experimental model of T-cell driven colitis was induced by the adoptive transfer of T cell from healthy WT BALB/c donor mice into Rag1^(−/−)^ recipient mice, that were either supplemented with or without recombinant Gal-9 (3 µg/ml, day 1, 3 & 5 intraperitoneal). (B) Changes in body weight (expressed as percentage of initial weight) were measured over the course of 28 days. (C) Dextran permeability (D) clinical score, (E) colon length were measured throughout the 28 days post transfer, and (F) representative photographs of colons isolated from mice were taken at the experimental endpoint. (G) Adoptive transfer of T cells from Gal-9 KO BALB/c vs WT BALB/c donor mice into recipient Rag1^(−/−)^ mice was also observed followed by measurement of (H) changes in body weight. Data are represented as means ± S.D (n = 3–10). Statistical analysis was performed using two-way ANOVA followed by Dunnett’s for multiple comparisons post-hoc test; *P ≤ 0.05, ^**^P ≤ 0.01, ^***^P ≤ 0.001, ^****^P ≤ 0.0001.

Inflammation in IBD is generally considered to be largely driven by disrupted T cell homeostasis, particularly CD4^+^ T cells. Therefore, we investigated the effect of Gal-9 specifically in a T cell-driven model of colitis, which the DSS model cannot truly capture as it is primarily driven by the innate immune system in the acute phase [45]. This was achieved by the adoptive transfer of naïve CD4^+^ T cells into recipient Rag^(-/-)^ mice, which induced severe intestinal inflammation (Fig. 3A). This was accompanied by decreased body weight, increased intestinal permeability denoted by FITC-dextran fluorescence, reduced colon length/inflammation and higher overall clinical scores: all of which are characteristic features associated with IBD. In contrast to the DSS-induced model of colitis, here the addition of rGal-9 (3 µg) demonstrated reduced intestinal inflammation as we observed a significant improvement in all disease parameters relative to T cell only transfer mice (Fig. 3 B-F). This highlights that exogenous Gal-9 may elicit responses distinct from its endogenous form, with effects that are highly context dependent. During intestinal inflammation, exogenous Gal-9 appears to exert anti-inflammatory effects, primarily targeting T cells. This was further confirmed by the adoptive transfer of Gal-9 KO T cells in recipient Rag^(-/-)^ mice whereby the lack of endogenous Gal-9 failed to improve body weight after a 40-day period (Fig. 3G, H).

### 3.3 Gal-9 deficiency did not alter murine Bone marrow derived macrophage function

**Fig. 4:**
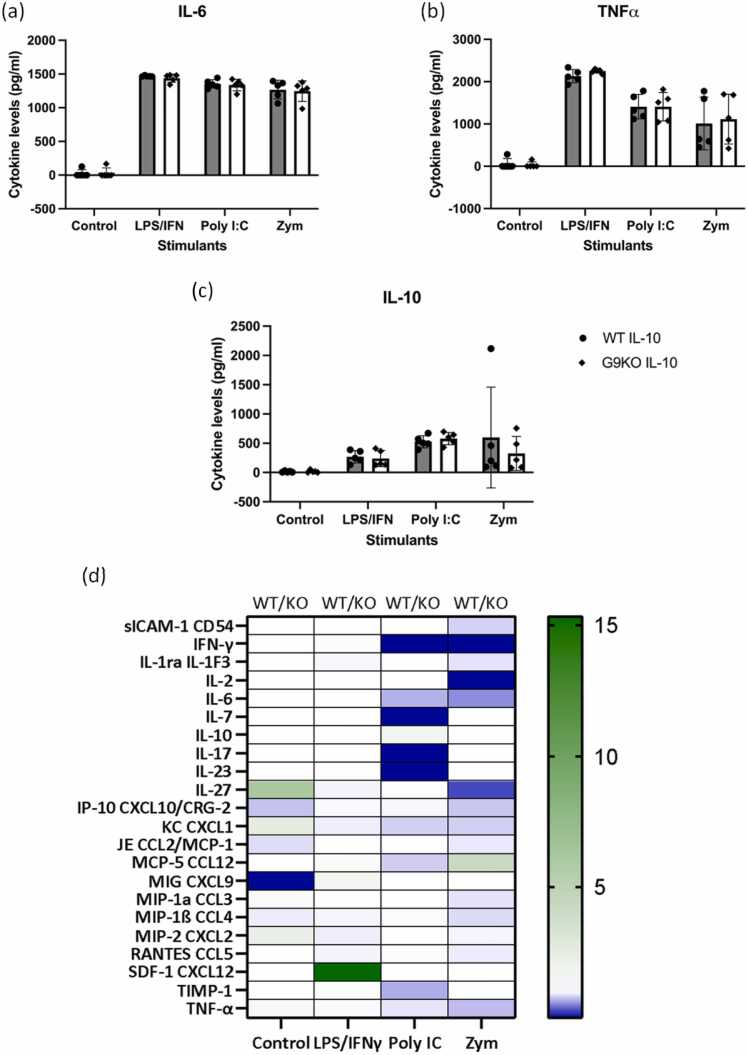
**Gal-9 deficiency did not alter murine bone marrow-derived macrophage function.** Examination of the impact of Gal-9 deficiency on cultured BMDMs in response to stimulation with LPS (100 ng/ml)/IFN (20 ng/ml) PolyI:C (25 μg/ml) and zymosan 10 μg/ml for 16 hrs. Culture supernatants were analysed by ELISA to measure cytokine levels for (A) IL-6, (B) TNFα and (C) IL-10. Supernatants were also pooled for multiplex cytokine analysis using Proteome Profiler™ protein array for WT or Gal-9KO BMDM with or without stimulation. Relative levels in WT compared to KO have been displayed in the double gradient heatmap. {Key: white = equivalent concentrations; green = higher in WT; blue = higher in Gal-9. Statistical analysis was performed using two-way ANOVA followed by Sidak for multiple comparisons post-hoc test; Data are represented as means ± S.D (n = 3–10) and considered significant at *P ≤ 0.05.

Macrophages are also essential drivers of intestinal inflammation during IBD, where they adopt a hyperactive, pro-inflammatory phenotype, in comparison to their anti-inflammatory quiescent nature during homeostasis. Both in CD and UC, macrophages exacerbate disease progression via antigen presentation and T cell mediated inflammation [28], [46]. Hence, we also investigated the effect of endogenous Gal-9 in murine bone marrow-derived macrophages (BMDM) in response to pattern-associated molecular patterns (PAMPs). BMDMs were stimulated with PAMPs including LPS/IFNγ, poly IC and zymosan and their response was assessed by performing a cytokine array on culture supernatants. Gal-9 deficiency did not alter BMDM responses since virtually identical responses to LPS/IFNγ, poly IC or zymosan stimulation were observed by cells isolated from WT and Gal-9KO mice. The results showed no detectable differences in the levels of most cytokines detected in the proteomic array including the classical inflammation-modulating cytokines; TNFα, IL-6 and IL-10 in both groups of mice (Fig. 4 A-D). These results suggest that the anti-inflammatory effects of endogenous Gal-9 are specific to T cells rather than macrophages.

### 3.4 Exogenous Gal-9 treatment induces pro-inflammatory macrophage phenotype

**Fig. 5:**
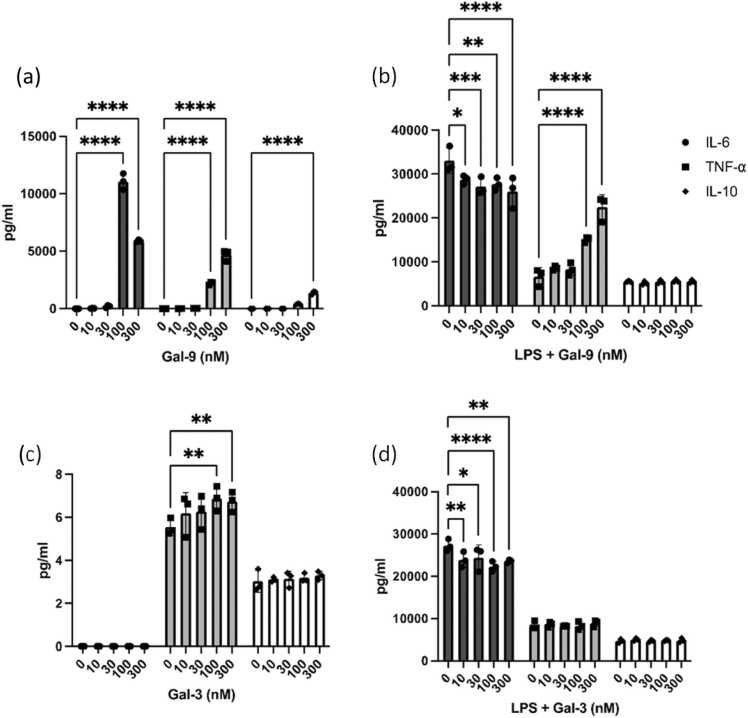
**Exogenous Gal-9 alters human macrophage function (cytokine profile).** The effect of varying concentrations of exogenous Gal-3 and Gal-9 (0–300 nM), with and without LPS (100 ng/ml) (16hrs) stimulation on cultured human macrophages was assessed by examining alterations in cytokine release. On day 7 of culture, supernatants were assessed for (A) IL-10, (B) TNFα and (C) IL-6 via Luminex assay. Statistical analysis was performed using one-way ANOVA with Dunnett for multiple comparisons post-hoc test; *P ≤ 0.05, ^**^P ≤ 0.01, ^***^P ≤ 0.001, ^****^P ≤ 0.000.

Further to this, we similarly repeated the previous experiment on human monocyte-derived macrophages (MDM), this time with the addition of exogenous Gal-9 and Gal-3 in response to LPS stimulation. Here we observed that exogenous Gal-9 alone stimulated TNF-ɑ release from human MDMs at concentrations of 100 nM and 300 nM respectively (Fig. 5A). When stimulated in combination with LPS, Gal-9 synergistically increased LPS-induced TNF-ɑ secretion in a dose-dependent manner, although no detectable differences were observed with Gal-3 (Fig. 5B). Moreover, Gal-9 stimulation in combination with LPS demonstrated a modest reduction in IL-6 levels with no differences observed in IL-10 secretion (Fig. 5B and D), whilst stimulation with Gal-3 alone and in combination with LPS showed little to no changes in cytokine release. Collectively, this data indicates that exogenous Gal-9 can modulate inflammatory responses in human MDMs, where they may be driving a pro-inflammatory phenotype.

## 4 Discussion

This study delves into the complex actions of Gal-9 in modulating disease progression and severity in murine models of IBD. Galectins, known for their functionality as both damage-associated molecular patterns (DAMPs) and receptors for PAMPs, are gaining increased attention for their multifaceted effects in inflammation [47], [48]. Using two distinct models of colitis - chemical dextran sulphate sodium (DSS)-induced injury, and a T-cell driven adoptive transfer model - we investigated the dichotomous effects of Gal-9 deficiency, as well as the impact of exogenous Gal-9 treatment. Our findings reveal a nuanced interplay between Gal-9 expression and disease outcomes providing a deeper understanding into the underlying pathophysiology of IBD and highlighting the potential of immunomodulation as a therapeutic strategy.

Our results demonstrate opposing effects of Gal-9, exhibiting both pro-inflammatory and pro-resolving phenotypes with distinct cell-specific effects, particularly on T-cells and macrophages. Gal-9 multifunctionality is evident in its diverse effects across inflammatory and autoimmune diseases, where its function is highly context-dependent and influenced by the cellular microenvironment [49], [50], [51]. For instance, in RA, Gal-9 has been shown to modulate inflammation by suppressing Th17 cell activity, a key driver of RA pathogenesis, while simultaneously supporting the expansion of Tregs and inducing apoptosis in activated T cells. These actions collectively reduce inflammation and joint damage in RA models [22], [52]. In models of atherosclerosis, Gal-9 deficiency reduced plaque burden and macrophage content, while exogenous Gal-9 enhanced efferocytosis by phagocytes causing reduced inflammation [50], [53], [54]. These findings underscore the importance of understanding the specific conditions and mechanisms through which Gal-9 operates, which could inform tailored therapeutic interventions for immune-mediated inflammatory diseases.

In the context of IBD, our study demonstrates that systemic Gal-9 deficiency mitigates, but does not entirely resolve markers of DSS-induced inflammation. Similar trends were observed for Gal-3, albeit to a lesser extent, suggesting potential pro-inflammatory effects for both Gal-9 and Gal-3 in intestinal inflammation. The use of global KO models, however, presents significant limitations. By eliminating the target gene across all tissues, global KO obscure tissue-specific effects of Gal-3 and Gal-9, particularly in the dynamic environment of the intestinal mucosa, where epithelial cells, immune cells, and endothelial cells interact.

Interestingly, supplementation with rGal-9 or rGal-3 protein did not modulate DSS-induced colitis severity, contrasting with previous studies reporting reduced colonic inflammation and disease severity [55]. These discrepancies may arise from variations in dose, binding of galectins intra- or extracellularly, structural integrity of galectins, glycosylation states, or the inflammatory microenvironment. The differential effects of exogenous galectins highlight the complexity of galectin biology and emphasize the need for further research to unravel context-specific mechanisms. For instance, Gal-9 has been shown to increase expression of its inhibitory receptor Tim-3 in mice with colitis induced by 2,4,6-trinitrobenzene sulfonic acid (TNBS) and DSS [56], [57]. Consistent with our findings, Gal-9 did not alter DSS-induced colitis severity, but alleviated TNBS induced colitis by inhibiting the TLR4/NF-κB pathway underscoring its context-dependent effects [56].

Given the accumulation of pro-inflammatory T-cells in the intestine as a hallmark of IBD, we further investigated the effect of Gal-9 using a T-cell-driven model of colitis. In T cell adoptive transfer models, a well-established approach for studying murine intestinal inflammation, we assessed disease severity using parameters such as weight loss, clinical score, and intestinal permeability [58], [59], [60]. Exogenous Gal-9 supplementation significantly reduced weight loss, improved intestinal barrier integrity, and lowered clinical scores, suggesting an anti-inflammatory, pro-resolution effect. Gal-9 deficiency in donor T cells, however, had minimal impact on disease severity. One of the key limitations of our study is the lack of detailed mechanistic evidence to elucidate the downstream effects of Gal-9 treatment on immune cells. While our findings suggest that Gal-9 modulates the immune response, the precise mechanisms through which this occurs remain unclear. Gal-9 is known to interact with Tim-3, which induces apoptosis in Th1 and Th17 cells and promotes Treg expansion, thereby enhancing immune tolerance and inflammation resolution [22], [61], [62], [63]. These processes can have profound immunomodulatory effects, potentially altering the balance between pro-inflammatory and anti-inflammatory responses. In addition to its effects on T cells, Gal-9 may influence other immune cell populations, such as macrophages, dendritic cells, and natural killer (NK) cells, either directly or indirectly. To address these gaps, future studies are needed to systematically investigate the downstream signalling pathways and cellular targets of Gal-9. Approaches such as single-cell RNA sequencing, proteomics, and functional assays in immune cell subsets will be instrumental in unravelling the comprehensive immunological effects of Gal-9.

Gal-9 effects, however, are highly context-dependent and influenced by its binding partners. For example, neutrophil-derived Gal-9 binding to CD44 on T cells enhances T-cell activation highlighting its dual effects in either stimulating or suppressing immune responses [64]. Beyond T cells, Gal-9 interacts with other immune populations, including neutrophils, NK cells, and macrophages, further underscoring its complex effects in inflammation. For instance, Gal-9 acts as a direct capture and activation receptor for neutrophils through β2 integrin and CD44-dependent mechanisms [65]. It also enhances NK cell effector functions by promoting MAPK signaling and IFNγ production [66]. Furthermore, we have previously shown that Gal-9 modulates platelet aggregation and activation via GPVI interactions, showcasing its broader immunomodulatory effects [67].

In innate immunity, Gal-9 exhibits pro-inflammatory effects [63]. Elevated plasma Gal-9 levels in COVID-19 patients, for instance, correlate with increased TNF-α and IL-6 expression in monocytes stimulated with rGal-9. Recombinant Gal-9 also induces activation markers (CD14, CD163) and inflammatory cytokines in monocytes and macrophages, driving innate immune-mediated inflammation [63], [68]. Similarly, in our study, exogenous Gal-9 exacerbated LPS-induced TNF-α secretion in human monocyte-derived macrophages (MDMs), while Gal-9 stimulation alone also induced pro-inflammatory mediator production, reinforcing its dualistic role in immune regulation.

The dichotomous effects of Gal-9 is not unprecedented, as similar findings have been frequently reported in the context of cancer. In such cases, Gal-9 has been shown to both enhance and inhibit tumour activity depending on its interactions with ligands expressed on T cells or innate immune cells [69]. The differences we observed between the functions of endogenous Gal-9 in the DSS model, and the responses induced by exogenous Gal-9, both in vitro and in vivo, may stem from the distinct behaviours of Gal-9 when acting intra- or extracellularly [10]. This suggests that the effects of exogenously added recombinant Gal-9 may not accurately represent the physiological effects of the endogenous protein. Further investigations into the mechanisms underlying this dichotomy are warranted to better understand how Gal-9 functions in these different contexts.

Gal-3 is widely recognised for its robust pro-inflammatory properties and is frequently elevated in the plasma of patients with various inflammatory conditions, yet our study presents a nuanced perspective [10], [70], [71]. Gal-3 exhibited no significant induction of TNF-α or IL-6 in MDMs, either alone or in combination with LPS, under our experimental conditions. This lack of pro-inflammatory activity underscores the context-dependent nature of Gal-3, which varies with cell type and experimental conditions. These findings highlight the distinct yet overlapping effects of Gal-9 and Gal-3 in immune modulation and the need to consider the cellular environment in assessing their contributions to inflammation.

Targeting Gal-9 therapeutically presents challenges due to its receptor promiscuity, where modulating one pathway risks unintended effects on others and potential disruption of immune homeostasis [72]. Systemic administration heightens the risk of off-target effect. Advanced delivery strategies, such as nanoparticle-based systems or antibody-conjugated therapies, may improve selectivity, but dosing complexities persist. High doses may induce immune exhaustion via prolonged TCR interaction, T-cell apoptosis, or excessive cytotoxic function suppression [73], [74], [75]. While neutralizing Gal-9 may enhance T and NK cell functionality for anti-tumor immunity, it risks pathological inflammation in autoimmune diseases or transplant rejection [74]. Long-term administration may also lead to compensatory pathway upregulation (e.g., PD-1/PD-L1, TIGIT) and desensitization of Gal-9 signalling [76]. Additionally, rGal-9 must be evaluated for safety, as it may elicit anti-drug immune responses, including antibody formation and hypersensitivity.

In summary, this study underscores the dichotomous effects of Gal-9 in inflammation particularly in IBD. The context-specific effects of Gal-9 on innate and adaptive immune cells highlight its potential as a therapeutic target, while also underscoring the challenges in achieving precise immunomodulation. These findings contribute to our understanding of Gal-9's immunoregulatory functions and support the development of novel therapeutic strategies to mitigate inflammatory disease burden.

## Author Contributions

ST, ASav, AF,JB, AAM, NM, ASch, JB, PT and JB performed experiments;. FM, DRK,VS and AJI designed the research; JB,AF.ST,AJI,DRK,FM wrote the manuscript. All authors read and approved the final manuscript. All authors have participated sufficiently in the work and agreed to be accountable for all aspects of the work

## Funding

This work is supported by a funding from F. Hoffmann-La Roche Ltd, Birmingham Fellowship to AJI, a Pfizer I-CRP project, and in part by a collaborative agreement between *ImmunoPharmaLab* at the Department of Pharmacy, University of Naples Federico II and the 10.13039/501100000855University of Birmingham (n. 2794224 Collaboration Agreement “ImmunoPEP”). ASav is supported by RTD-A research contract for the thematic spoke "Validating acid nucleic-based drugs using *in vitro* and *in vivo* models of cancer and immune-related diseases". The research activity is focused on topics of interest for the Department of Pharmacy, Federico II, included in the grant application marked MUR identification code CN00000041 "National Center for Gene Therapy and Drugs based on RNA Technology” (initiative financed by the 10.13039/501100000780European Union – NextGenerationEU and Funding granted with Directorial Decree n.1035 of 06.17.2022 under PNRR MUR – M4C2 – Investment 1.4- CUP UNINA: E63C22000940007). NM is supported by AORN A. Cardarelli Scholarship (n. 725/2022 GRC- Linea Progettuale 1.3: “Gestione delle cronicità”). ASch is supported by University of Naples Federico II PhD scholarship in “Nutraceuticals, functional foods and human health” (PNRR DM 118 M4C1– INV 4.1 ricerca PNRR generici). AJI Supported 10.13039/501100000274BHF Project grant PG/23/11476. AAM extends his appreciation to the Deanship of Research and Graduate Studies at 10.13039/501100007446King Khalid University for funding this work through small group research project under grant number RGP1/122/45.

## CRediT authorship contribution statement

**Schettino Anna:** Writing – review & editing, Investigation, Formal analysis, Data curation. **Blaising Julie:** Writing – review & editing, Investigation, Formal analysis. **Trenkle Patrick:** Writing – review & editing, Investigation, Formal analysis, Data curation. **Sandrin Virginie:** Writing – review & editing, Writing – original draft, Supervision, Conceptualization. **Maione Francesco:** Writing – review & editing, Supervision, Investigation, Formal analysis, Data curation. **Regan-Komito Daniel:** Writing – review & editing, Writing – original draft, Supervision, Project administration, Conceptualization. **Tull Samantha:** Writing – original draft, Investigation, Formal analysis, Data curation. **Iqbal Asif Jilani:** Writing – review & editing, Writing – original draft, Funding acquisition, Conceptualization. **Saviano Anella:** Writing – review & editing, Investigation, Formal analysis, Data curation. **Fatima Areeba:** Writing – review & editing, Writing – original draft, Investigation, Formal analysis. **Begum Jenefa:** Writing – review & editing, Writing – original draft, Investigation, Formal analysis. **Abo Mansour Adel:** Writing – review & editing, Investigation, Formal analysis, Data curation. **Marigliano Noemi:** Writing – review & editing, Investigation, Formal analysis.

## Declaration of Competing Interest

The authors declare the following financial interests/personal relationships which may be considered as potential competing interests: Asif Jilani Iqbal reports financial support was provided by F Hoffmann-La Roche Ltd. Asif Jilani Iqbal reports financial support was provided by British Heart Foundation. Adel Abo Mansour reports financial support was provided by King Khalid University. If there are other authors, they declare that they have no known competing financial interests or personal relationships that could have appeared to influence the work reported in this paper.
